# The impact of nonpsychotic postpartum psychiatric disorders (NPPDs) on offspring

**DOI:** 10.1192/j.eurpsy.2023.1522

**Published:** 2023-07-19

**Authors:** J. Stojanov, M. Stanković, A. Stojanov

**Affiliations:** 1 Service for acute psychotic disorders, Special Hospital for Psychiatric Disorders, Gornja Toponica, Serbia; 2 3. Center of Mental Health Protection, Clinical Centre Nis, Nis, Serbia, 2. Faculty of Medicine, University of Nis, Nis, Serbia; 3 4. Clinic of Neurology, Clinical Center Nis, Nis, Serbia

## Abstract

**Introduction:**

Non-psychotic postpartum psychiatric disorders (NPPDs) are among the most common underdiagnosed mental disorders with a preserved reality test after delivery.

**Objectives:**

NPPDs have been shown to have an association with infant growth, attachment, sleep, temperament and ultimately offspring’s emotional, behavioural, cognitive and social development.

**Methods:**

Most prevalent NPPDs are postpartum mood and anxiety disorders, as well as obsessive–compulsive disorder, post-traumatic stress disorder and eating disorders.

**Results:**

The high methodological quality of the reviewed studies strengths the association between NPPDs and different disorders in the neurodevelopmental period with a negligible impact on mental status in adolescence and adulthood. NPPDs showed an effect on offspring’s emotional, behavioural, cognitive and social development, due to common developmental mechanisms.

**Conclusions:**

Timely accurate identifying and treating NPPDs, by using NPPDs symptoms screening tools could reduce the incidence of mental disorders in offspring. Although neurodevelopmental disorders and mental disorders related to pregnancy as separate diagnostic categories have been insufficiently researched, the potential impact of postpartum mental disorders on children’s development is an extremely unexplored field that should be focused on in further scientific research.

**Disclosure of Interest:**

None Declared

